# Side-to-side asymmetries in landing mechanics from a drop vertical jump test are not related to asymmetries in knee joint laxity following anterior cruciate ligament reconstruction

**DOI:** 10.1007/s00167-017-4651-2

**Published:** 2017-07-15

**Authors:** Christophe A. G. Meyer, Paul Gette, Caroline Mouton, Romain Seil, Daniel Theisen

**Affiliations:** 1 Sports Medicine Research Laboratory, Department of Population Health, Luxembourg Institute of Health, 76, Rue d’Eich, 1460 Luxembourg, Luxembourg; 2 Department of Orthopaedic Surgery, Centre Hospitalier de Luxembourg – Clinique d’Eich, Luxembourg, Luxembourg

**Keywords:** Knee injury, Knee kinematics, Knee kinetics, Static anterior laxity, Rotational knee laxity, Asymmetrical knee loading

## Abstract

**Purpose:**

Asymmetries in knee joint biomechanics and increased knee joint laxity in patients following anterior cruciate ligament reconstruction (ACLR) are considered risk factors for re-tear or early onset of osteoarthritis. Nevertheless, the relationship between these factors has not been established. The aim of the study was to compare knee mechanics during landing from a bilateral drop vertical jump in patients following ACLR and control participants and to study the relationship between side-to-side asymmetries in landing mechanics and knee joint laxity.

**Methods:**

Seventeen patients following ACLR were evaluated and compared to 28 healthy controls. Knee sagittal and frontal plane kinematics and kinetics were evaluated using three-dimensional motion capture (200 Hz) and two synchronized force platforms (1000 Hz). Static anterior and internal rotation knee laxities were measured for both groups and legs using dedicated arthrometers. Group and leg differences were investigated using a mixed model analysis of variance. The relationship between side-to-side differences in sagittal knee power/energy absorption and knee joint laxities was evaluated using univariate linear regression.

**Results:**

A significant group-by-leg interaction (*p* = 0.010) was found for knee sagittal plane energy absorption, with patients having 25% lower values in their involved compared to their non-involved leg (1.22 ± 0.39 vs. 1.62 ± 0.40 J kg^−1^). Furthermore, knee sagittal plane energy absorption was 18% lower at their involved leg compared to controls (*p* = 0.018). Concomitantly, patients demonstrated a 27% higher anterior laxity of the involved knee compared to the non-involved knee, with an average side-to-side difference of 1.2 mm (*p* < 0.001). Laxity of the involved knee was also 30% higher than that of controls (*p* < 0.001) (leg-by-group interaction: *p* = 0.002). No relationship was found between sagittal plane energy absorption and knee laxity.

**Conclusions:**

Nine months following surgery, ACLR patients were shown to employ a knee unloading strategy of their involved leg during bilateral landing. However, this strategy was unrelated to their increased anterior knee laxity. Side-to-side asymmetries during simple bilateral landing tasks may put ACLR patients at increased risk of second ACL injury or early-onset osteoarthritis development. Detecting and correcting asymmetric landing strategies is highly relevant in the framework of personalized rehabilitation, which calls for complex biomechanical analyses to be applied in clinical routine.

**Level of evidence:**

III.

## Introduction

Patients undergoing anterior cruciate ligament reconstruction (ACLR) have an increased risk of re-injury when returning to sport activities, especially in the first two years following surgery [28, 39]. Previous studies have suggested that this increased risk of subsequent ipsilateral or contralateral ACL tear might be related to abnormal knee function during dynamic movements [9, 29, 30]. In addition, since abnormal knee biomechanics have been suggested as a contributing factor to knee osteoarthritis development [2, 6, 12], their assessment is crucial in the evaluation of functional recovery. The current literature highlights landing mechanics evaluation during standardized jump tasks as an interesting screening tool for injury risk. Several studies have reported asymmetric landing strategies among patients following ACLR [4, 27, 29]. Paterno et al. [29] reported increased knee range of motion in the frontal plane as well as increased knee flexion moment at initial contact during landing from a drop vertical jump at the injured compared to the non-injured leg. These alterations were suggested as predictive factors of subsequent ACL injury in patients after ACLR. Similarly, Oberländer et al. [26] found that patients following ACLR had persistent abnormal knee biomechanics at the injured leg during a single-leg hop test, which may place them at higher risk of knee osteoarthritis development. The observed asymmetrical biomechanical pattern has often been proposed to arise from quadriceps strength deficits in the involved leg [16, 31], patients with the greatest deficits showing the largest asymmetries in sagittal knee biomechanics. The link between muscle strength deficits and abnormal biomechanics has led the scientific community to investigate joint energetics, as represented by the physical work performed by the muscles. Indeed, the integration of the joint mechanical power curve over time, i.e. work, is an indirect measure of muscle work giving insights into the movement strategy [40]. Nonetheless, few studies have investigated mechanical work during landing in the context of ACLR.

Another important aspect related to knee function following ACLR is static knee joint laxity [1, 5, 14, 19]. In spite of surgery seeking to restore normal knee laxity, greater anteroposterior, but not rotational, knee joint laxity has been shown in patients following ACLR [19]. Additionally, increased static knee joint laxity has been associated with greater risk of primary ACL injury [23, 36].

Static knee joint laxity measures represent an analytical evaluation of the passive knee joint characteristics. It is currently not known in how far the latter impact knee joint control under dynamic conditions. Indeed, the relationship between knee laxity and dynamic knee biomechanics has not yet been analysed in the context of patients following ACLR. The existence of such relationship would have implications for both surgical and rehabilitative interventions. To the authors’ knowledge, only two research groups have investigated this topic in a healthy population [33–35]. Shultz et al. [33] found that increased knee joint laxity was associated with greater knee valgus angles and internal knee varus moments during landing. Furthermore, increased anterior knee joint laxity was associated with greater knee energy absorption, but only in females [34]. Torry et al. [35] found that anterior knee laxity was related to peak anterior tibial translation during stiff drop landings. How far these relationships apply to ACLR patients remains to be determined.

The aim of this study was therefore to investigate knee landing mechanics during a drop vertical jump task in patients following ACLR and in control participants. It was hypothesized that (1) ACLR patients would demonstrate greater side-to-side differences in knee landing biomechanics than controls, (2) ACLR patients would display greater side-to-side differences in knee joint laxity, and finally (3) asymmetries in knee landing mechanics would be related to asymmetries in knee joint laxity.

## Materials and methods

Thirty-one patients consulting at the orthopaedic department of our clinic between February and December 2015 met the inclusion criteria: age range 15–35 years, unilateral ACL injury, no other previous lower limb injuries that could affect jump performance, minimum of 6 months post-surgery, full knee extension, minimum of 140° knee flexion and medically cleared to perform the protocol tests. Eventually, 17 of these patients agreed to take part in our study. All had participated in sports before surgery (Table 1) and had an average Knee injury and Osteoarthritis Outcome Score (KOOS) for sports and recreation of 83 (±15) (self-administered questionnaire) at the time of testing.

**Table 1:** Demographics of study participants

	Controls (*n* = *28*)	Patients (*n* = *17*)
Age (years)	25.4 (±4.1)	24.5 (±6.8)
BMI (kg m^−2^)	22.4 (±1.9)	23.1 (±2.4)
Gender (females/males)	14F/14 M	5F/12 M
Follow-up time (months)	NA	8.9 (±1.3)
ACL injury mechanism	NA	4 contact/13 non-contact
Graft type	NA	11 HS/6 BPTB
Associated lesions^a^	NA	Yes (*n* = 11)/No (*n* = 6)
Sports participation (h/week)	5 (±2)	8 (±4)
Sport activity level^b^	I (*n* = 10); II (*n* = 7); III (*n* = 11)	I (*n* = 13); II (*n* = 2); III (*n* = 2)

Data are presented as means (±SD)

*HS* hamstring graft, *BPTB* bone–patellar tendon–bone graft (note: three patients underwent extra-articular lateral tenodesis)

^a^ Associated lesions were medial meniscal tear (*n* = 1), lateral meniscal tear (*n* = 4) or both (*n* = 6)

^b^ Sport activity level before injury: level I refers to sports with jumping, pivoting, hard cutting actions (e.g. football, basketball, handball), level II refers to moderate pivoting, jumping and cutting actions (e.g. skiing, tennis, volleyball), and level III refers to sports with no pivoting actions (jogging, running) [13]

A control group including twenty-eight healthy, active, age-matched volunteers was selected. Their recruitment was based on questionnaires to exclude any previous knee injury, musculoskeletal and neurological disorders and any impairment interfering with the task. Knee pain during task execution was monitored and used as an exclusion criterion for all participants.

### Drop vertical jump test

Participants were familiarized with the experimental procedures during a separate test session. The protocol consisted in executing several drop vertical jumps (DVJs) during which three-dimensional impact forces and lower limb movements were measured. Jump tasks were preceded by a 10-min warm-up run on a treadmill at a self-selected pace. Participants wore tight clothing and standardized footwear during testing.

**Fig. 1:**
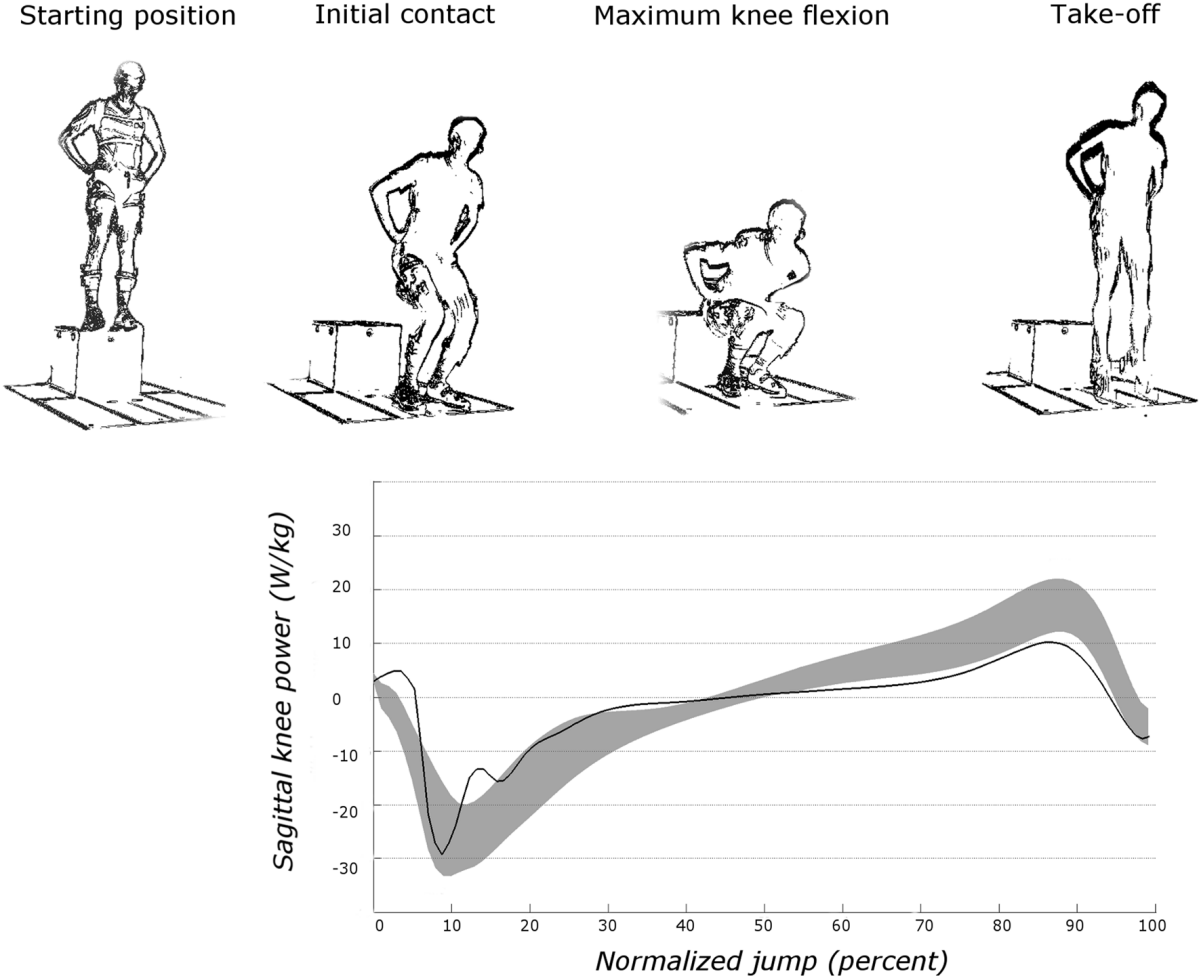
Schematic representation of the drop vertical jump test. In the lower part, a typical graphical representation of sagittal plane knee power is represented. The grey zone represents average ±1SD of the control group

DVJs were performed from a height of 0.4 m, with arms akimbo and feet hip width apart. Participants were instructed to drop off a box, land with their feet on separate force platforms and perform a maximal vertical jump after the first landing (Fig. 1). Participants performed several DVJs until three valid trials were recorded. Trials were considered invalid when participants jumped from the box instead of dropping or lost their balance, when their hands did not remain on the waist or when the feet did not touch separate force platforms.

**Fig. 2:**
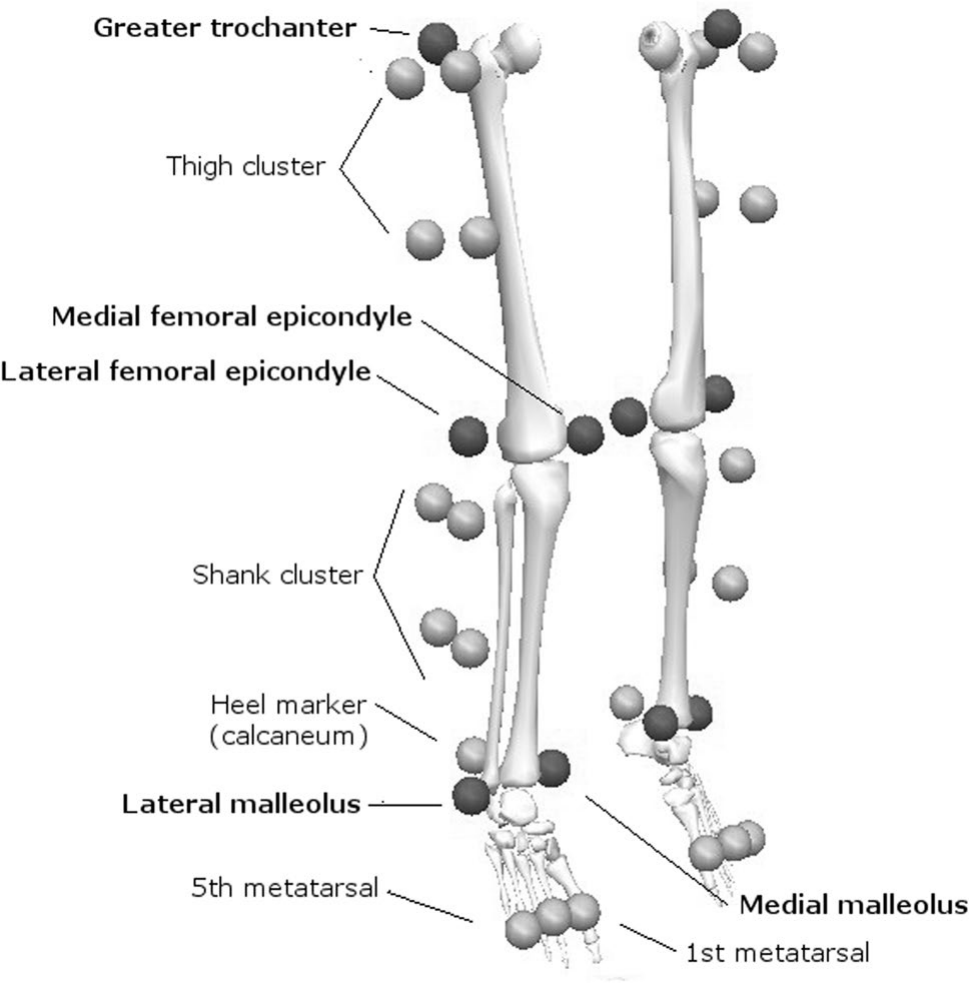
Marker positions defining lower limb kinematic model. Markers indicated in bold are anatomical markers that were used during static calibration only

After recording height and mass, participants were fitted with 34 active markers according to a 6-degrees-of-freedom six-segment lower limb model including feet, shanks and thighs. A single experienced assessor performed marker positioning to minimize placement errors. Tracking markers (*n* = 24) comprised rigid clusters of four markers applied on the thighs and shanks, as well as four markers attached on the shoes. Prior to dynamic testing, anatomical calibration of each segment and neutral limb alignment were defined using additional anatomical markers (*n* = 10) applied on the malleoli, femoral condyles and greater trochanter of each limb (Fig. 2). Three-dimensional marker trajectories were recorded using four CODA CX1 optoelectronic motion capture units (Charnwood Dynamics Ltd, Leicestershire, UK) sampling at 200 Hz. Ground reaction forces for each leg were synchronously collected at 1000 Hz using two separate force platforms (Arsalis 800 × 500; Arsalis SPRL; Louvain-la-Neuve, Belgium).

### Biomechanical data processing

Data processing was performed using Visual 3D (v5.02.24; C-Motion, Inc., Germantown, MD) and custom-made MATLAB program (MATLAB R2014a, The MathWorks^®^, Natick, MA, USA). Initial contact and take-off events were determined based on a 10 N threshold from the vertical ground reaction force vector. Kinematic and kinetic signals were low-pass-filtered using the same fourth-order Butterworth filter with a 6 Hz cut-off frequency [18]. Knee joint angles were calculated using a Cardan XYZ rotation sequence. Net external knee joint moments were computed using standard inverse dynamics. All data were time-normalized from 0 to 100% with respect to the contact phase of the landing. Biomechanical variables of interest were determined only during the eccentric landing phase defined as initial contact to maximal knee flexion angle.

Biomechanical variables were averaged from the three valid trials and included knee flexion and valgus angles at initial contact, peak knee flexion and valgus angles, net peak knee flexion, valgus and varus moments, and sagittal peak knee power absorption. Sagittal knee power was computed at each time point by multiplying the angular velocity of the sagittal knee angle with the sagittal knee joint moment [10]. Hence, the work done by the knee extensors during the phase of landing, i.e. energy absorption by the knee extensor muscles, was calculated by integrating the joint power curve over the time of the negative phase. All kinetic data were normalized to body mass.

### Knee joint laxity

**Fig. 3:**
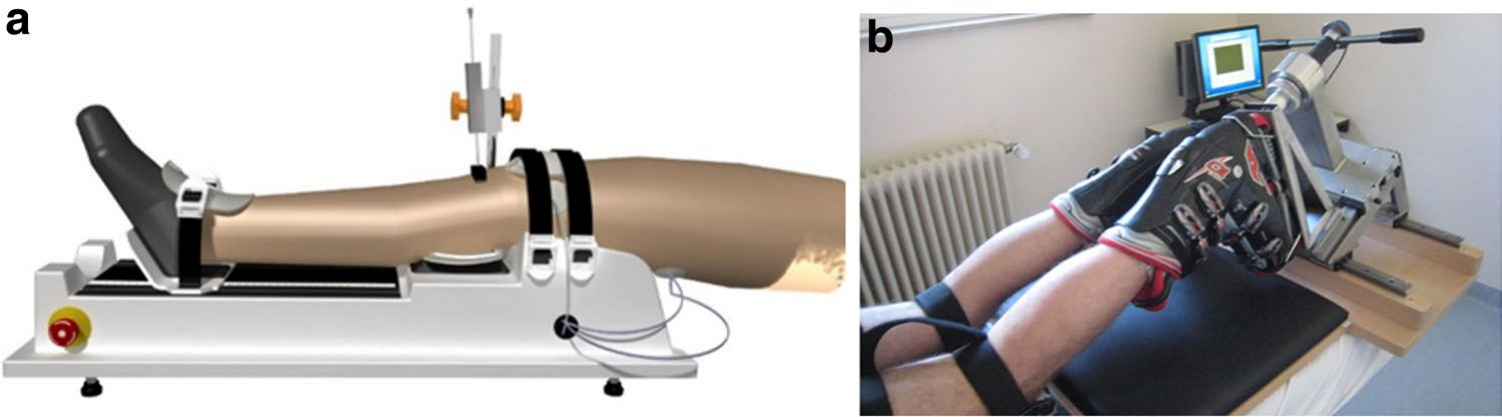
Illustration of the devices used for the evaluation of the knee joint laxity. A—leg of a participant on the GNRB for anterior knee joint laxity. B—leg of a participant for rotational knee joint laxity

All participants underwent anterior and internal rotation knee laxity evaluations on both legs using previously described procedures yielding a standard error of measurement of less than 10% [22] (Fig. 3). In patients, the non-injured knee was tested first, while the first knee tested in controls was randomly chosen. Anterior knee laxity measures were performed using the GNRB© arthrometer (GeNouRob company, France) allowing to perform a standardized Lachman test using a calibrated motor. Participants were supine with the tested leg on the device and the non-tested leg resting on the examination table. The tested knee was flexed at 20° and positioned with the femoral intercondylar line at the edge of the leg support. The knee was immobilized via a patella shell and positioned so as to ensure central alignment with the tibial axis. The foot was tightened on a foot support. Maximal anterior tibial displacement (ATD200) was measured by applying a progressive force up to 200 N via a plateau fixed on a hydraulic cylinder. Displacement was recorded to the nearest 0.1 mm by a sensor placed perpendicularly to the tibia on the tibial tuberosity. The average of the last two of three successive ATD200 measurements was taken for further calculations. Rotational knee laxity was evaluated in the internal direction using the rotameter device to reproduce a standardized dial test [21]. Patients were laying prone on an examination table with their thighs fixed, knee flexed at 30° and the foot immobilized in a ski boot, itself affixed to a manual handle bar. The latter was used to apply a progressive torque up to 5 Nm, and the resulting rotation (IR5) was measured via an inclinometer of an accuracy of 0.01°. The average of the last two of four successive IR5 measurements was taken for further data processing.

All procedures were approved by the National Research Ethics Committee (CNER, Luxembourg, approval n°201101/05). Written informed consent was obtained prior to participation.

### Statistical analysis

Based on preliminary analysis of 10 patients, we expected an effect size of 1.0 for the side-to-side difference in peak knee power and energy absorption during landing from a drop jump, our main variables of interest. Thus, given a power of 0.8 and an alpha level of 0.05, 16 patients were required to detect significant differences between the two legs.

For testing group and leg differences, a distinction was made between the involved (operated) (ACLR_inv_) and the non-involved leg (ACLR_*n*-inv_) in patients, while for controls the involved (CON_inv_) and non-involved legs (CON_*n*-inv_) were randomly chosen [38]. Thus, biomechanical variables were compared using a mixed model analysis of variance [group (2) × leg (2)]. When a significant group-by-leg interaction was found, a two-tailed post hoc comparison (between limbs and between groups) was carried out. Data are presented as average ± standard deviation (SD). Statistical significance was set at *p* < 0.05. The side-to-side differences, expressed as the difference between the involved and non-involved leg, were determined for the biomechanical variables as well as for the laxity data. To study the relationship between peak knee power/energy absorption and knee laxity, univariate linear regressions were computed. All statistical analyses were performed using SPSS Version 23 (IBM, Houston, TX, USA).

## Results

Table 1 summarizes participants’ characteristics. ACLR patients were evaluated at an average of 8.9 ± 1.3 months (median 8.6; range 6–11) after surgery.

While no group-by-leg effect was found for peak knee power absorption in the sagittal plane during landing from the DVJ (n.s), a group-by-leg interaction (*p* = 0.010) was found for knee energy absorption (Table 2). Post hoc analysis revealed that ACLR_inv_ had 25% lower knee energy absorption than ACLR_*n*-inv_ (*p* < 0.001), as well as 18% lower than CON_inv_ (*p* = 0.018). In 14 patients, the side-to-side differences (ACLR_inv_–ACLR_*n*-inv_) in sagittal plane energy absorption at the knee were outside the 95% confidence interval of differences found for the control group. These strong asymmetries were negative in 11 patients (69%), illustrative of an unloading strategy of their involved leg, while they were positive in three patients, showing overloading of the involved leg.

**Table 2:** Biomechanical variables for controls and patients at both legs during landing from a drop vertical jump

Variables	Controls (*n* = *28*)		ACLR patients (*n* = *17*)		*p*-values		
	Involved	Non-involved	Involved	Non-involved	Leg effect	Group effect	Leg*group
Peak knee power absorption^a^ (W kg^−1^)	15.07 (±3.11)	16.31 (±2.70)	14.11 (±4.15)	17.31 (±3.38)	0.003	n.s	n.s
Energy absorbed^b^ (J kg^−1^)	1.49 (±0.32)	1.58 (±0.27)	1.22 (±0.39)	1.62 (±0.40)	<0.001	n.s	0.010^§,†^
Knee flexion angle at initial contact (°)	26.5 (±9.5)	27.2 (±9.0)	23.4 (±8.0)	24.0 (±7.0)	n.s	n.s	n.s
Knee valgus angle at initial contact (°)	3.3 (6.3)	4.5 (4.7)	3.7 (4.9)	2.0 (3.4)	n.s	n.s	0.023
Peak knee flexion angle (°)	101.0 (±11.4)	101.7 (±11.8)	93.8 (±14.9)	96.9 (±14.7)	0.019	n.s	n.s
Peak knee valgus angle (°)	4.6 (±6.6)	6.1 (±5.0)	6.2 (±4.6)	5.3 (±4.9)	n.s	n.s	n.s
Peak knee flexion moment (Nm kg^−1^)	1.91 (±0.30)	2.03 (±0.26)	1.74 (±0.35)	2.10 (±0.44)	0.001	n.s	n.s
Peak knee valgus moment (Nm kg^−1^)	0.06 (±0.08)	0.07 (±0.08)	0.14 (±0.13)	0.17 (±0.10)	n.s	0.001	n.s
Peak knee varus moment (Nm kg^−1^)	0.36 (±0.27)	0.35 (±0.17)	0.17 (±0.10)	0.19 (±0.17)	n.s	0.001	n.s
Peak vertical GRF (×*BW*)	1.67 (±0.41)	1.74 (±0.46)	1.83 (±0.57)	1.87 (±0.47)	n.s	n.s	n.s
ATD200 (mm)	4.3 (±0.8)	4.3 (±0.6)	5.6 (±1.1)	4.4 (±0.8)	<0.001	0.002	0.002^§,†,‡^
IR5 (°)	19.24 (±5.10)	19.22 (±4.50)	20.35 (±4.80)	19.48 (±4.29)	n.s	n.s	n.s

Jumps are defined from touch down to maximal knee flexion

*GRF* ground reaction force, *BW* body weight, *ATD200* = maximal anterior tibial displacement at 200 Newton, *IR5* maximal rotational knee laxity at 5 Nm

^§^ Significant difference between ACLR_inv_ and ACLR_n-inv_

^†^ Significant difference between ACLR_inv_ and CON_inv_

^‡^ Significant difference between ACLR_*n*-inv_/CON_*n*-inv_

^a^ Peak knee power absorption in the sagittal plane

^b^ Energy absorbed in the sagittal plane

There was a main leg effect for peak knee flexion angle and peak external knee flexion moment, with overall greater values found for the non-involved legs in both groups. ACLR_inv_ had 17% lower peak knee flexion moment compared to ACLR_*n*-inv_, while only a 6% difference was found for CON_inv_ and CON_*n*-inv_. Significant main group effects (*p* = 0.001) were found for peak knee valgus and varus moments, which were higher and lower, respectively, in the patient group.

A group-by-leg interaction (*p* = 0.002) was found for ATD200 (Table 2), with a 27% higher value (*p* < 0.001) for ACLR_inv_ compared to ACLR_*n*-inv_, and a 30% higher result (*p* < 0.001) compared to both legs of the control group. A group effect was found (*p* = 0.002) for ATD200 illustrating the overall greater anterior knee laxity in the patient group (pooled difference = 0.7 mm). No significant group-by-leg interaction or main effect was found for IR5.

**Fig. 4:**
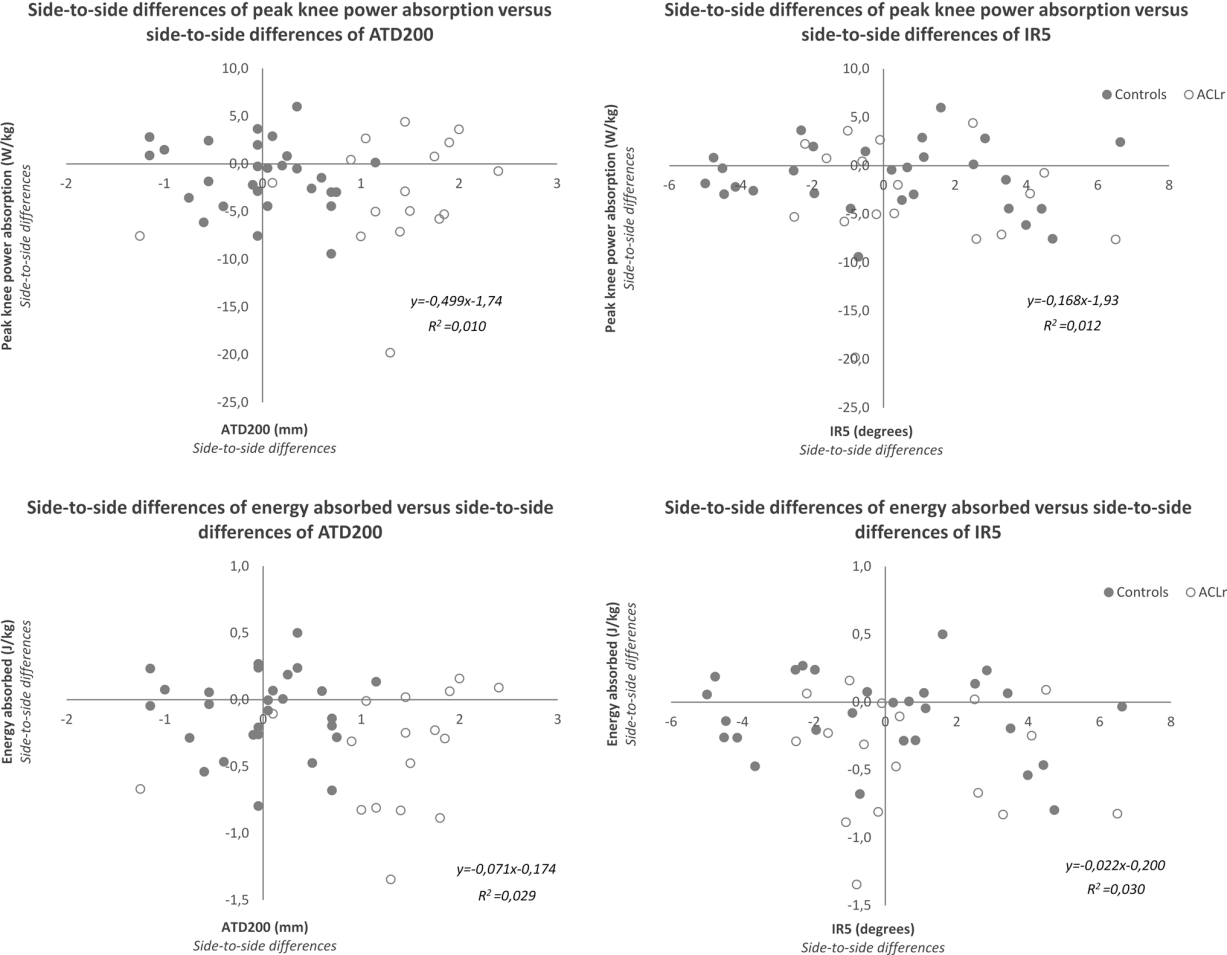
Scatter diagram displaying the relationship between biomechanical data and knee joint laxity. Scatter diagram displaying the relationship between side-to-side differences of knee joint laxity (abscissa axis) and side-to-side differences of knee power absorption and energy absorption (ordinate axis). *ATD200* maximal anterior displacement at 200 N, *IR5* maximal internal rotation at 5 Nm. Negative side-to-side differences in power and energy represent decreased values at the operated side

The graphical representation of the relationship between side-to-side differences in sagittal peak knee power absorption/energy absorption and knee laxity is depicted in Fig. 4. No relationship was found between peak knee power absorption and ATD200 (n.s), neither between energy absorbed and ATD200 (n.s). A similar observation was made for the relationship between peak knee power absorption and IR5 (n.s) and energy absorbed and IR5 (n.s).

## Discussion

The most important finding of the present study was that 9 months post-surgery, patients following ACLR showed asymmetry in their knee biomechanics during a bilateral landing task, which was unrelated to their side-to-side knee joint laxity differences. Our study is the first to compare ACLR patients with healthy control participants regarding both knee landing biomechanics/energetics and static knee joint laxity. While no significant differences were found for peak knee power absorption in the sagittal plane, patients displayed unbalanced knee energy absorption, with a lower performance recorded at their involved leg. The significant group-by-leg interaction and post hoc analyses confirm our hypothesis of greater side-to-side differences in knee landing biomechanics/energetics of ACLR patients compared to controls during bilateral landing. Furthermore, patients had greater anterior knee joint laxity at their involved leg compared to the uninvolved one or the control group, thus confirming our second hypothesis. However, no relationship was found between side-to-side differences in knee landing mechanics and anterior or rotational knee joint laxity, thus infirming our third hypothesis.

Decreased knee work absorption at the involved leg following rehabilitation suggests that ACLR patients tend to reduce the load on the reconstructed knee and to overload the uninvolved leg. This load-shifting strategy is also illustrated in our data of external peak knee flexion moment and power absorption, although the interactions were not significant. These findings corroborate previous research [25, 26, 31]. In a prospective cohort of 10 patients twelve months post-ACLR, Oberländer et al. [25] demonstrated lower sagittal peak knee flexion moments at the involved leg during single-leg landing. They reported a moment redistribution to the adjacent joints, with increased hip flexion and ankle plantarflexion moments. We speculate that our patients adopted a similar moment redistribution strategy during bilateral landing, given that peak GRF was similar in both legs (Table 2). The reason for these landing mechanics could be related to knee extensor muscle strength deficits [25, 31]. Our patients indeed presented with an average quadriceps strength deficit of 12% (range 1–25%), but quadriceps strength was not related to peak knee power absorption, energy absorption or peak knee flexion moment (Pearson’s product-moment correlations between −0.35 and 0.42; n.s.). Although not tested here, contributing factors of altered knee mechanics may include abnormal muscle activation [8, 24] or psychological aspects such as fear of loading or re-injury [3, 20].

Altered sagittal plane knee mechanics during landing might be indicative of increased risk of second ACL injuries or early-onset osteoarthritis, but frontal plane mechanics may also play a role [15, 29]. Although we found no differences in knee valgus angle between patients and control participants, a main group effect was present for peak knee valgus and varus moments. The greater peak knee valgus moments displayed by our patients could reflect greater valgus loading and higher risk of future injury. However, the values found here were about 5 times lower than those from a previous study [15]. In addition, patients globally displayed a reduced peak knee varus moment (0.18 ± 0.14 Nm kg^−1^) compared to controls (0.35 ± 0.22 Nm kg^−1^). This finding is in line with previous research on gait [37], but contrasts with the suggestion that greater knee external adduction moments could be related to early-onset medial tibiofemoral osteoarthritis [32]. Medial compartment knee osteoarthritis in patients following ACLR might result from other changes in knee mechanics and/or biological/anatomical changes [7, 17].

A previous study reported that ACLR patients displayed increased anterior but not rotational knee joint laxity at their operated leg 27 months following surgery [19]. The group-by-leg interaction, the main group and leg effects for ATD200 and the absence of differences for internal rotational laxity found here support these findings [19]. Our ACLR patients had a 1.3 mm greater ATD200 at their involved leg compared to their non-involved, which is in accordance with previous research [11, 19]. Such a side-to-side difference might play an important role in subsequent injury risk or further degeneration of the knee joint, as suggested by Myer et al. [23] after screening 1558 athletes for predictors of ACL injury. They reported a fourfold increase in the likelihood of ACL injury for a 1.3 mm side-to-side difference.

Whether differences in knee laxity are associated with neuromotor control has not been investigated in ACLR patients. A previous study [34] found that in females, but not in males, knee work absorption was positively correlated with knee joint laxity during a bilateral drop jump task. However, in our patients, no correlation between peak knee power or energy absorption and ATD200 was found, neither overall, nor in males and females separately (Pearson’s product-moment correlations between −0.28 and 0.22; n.s.). On the contrary, energy absorbed was 25% lower, while anterior laxity was 27% higher in the involved compared to the uninvolved leg. Again this observation holds true for females and males separately. Furthermore, the side-to-side differences in biomechanical variables illustrating this loading shift were unrelated to joint laxity differences (Fig. 4), irrespectively of sex.

One shortcoming of this study is that our focus was exclusively on the knee joint. Thus, we cannot provide a complete picture of the biomechanical compensation strategy adopted by the ACLR patients to unload their involved knee. Insofar, recommendations as to how to restore side-to-side symmetry are limited. Furthermore, our conclusions are based on bilateral landing from a drop jump and may not necessarily hold true for single-leg landing tasks that require greater neuromuscular control. Further study is also warranted regarding the association of knee biomechanics and static knee laxity during other jump tasks, as well as during activities of daily living. Confirmation that there is no relationship between active knee control and static joint laxity would imply that they are independent and complementary characteristics of knee function. In such a case, the sole evaluation of knee joint laxity would not be appropriate to describe knee function under dynamic conditions. Detailed biomechanical analyses should be preferred here to identify abnormal dynamic knee behaviour and form the basis for individualized rehabilitation strategies to be implemented in clinical practice.

## Conclusion

Nine months following surgery, ACLR patients demonstrated side-to-side biomechanical asymmetries in the knee joint in the sagittal, but not the frontal plane. These observations are illustrative of a general unloading strategy at the reconstructed leg. Side-to-side differences were also found in anterior knee laxity measurements, but these were not related to abnormal knee biomechanics during a drop vertical jump. Side-to-side asymmetries in general, and knee unloading in particular, during bilateral tasks as simple as landing from a drop vertical jump may be a risk factor for second ACL injury or early-onset knee osteoarthritis development in ACLR patients. The detection of such strategies bears high clinical relevance for these patients and may thus justify complex biomechanical analyses to be applied in clinical routine.
